# Cardiac arrest during vigorous exercise: coronary plaque rupture or myocardial ischaemia?

**DOI:** 10.1007/s12471-014-0647-4

**Published:** 2015-01-09

**Authors:** Alexander R. van Rosendael, Michiel. A. de Graaf, Arthur J. Scholte

**Affiliations:** 1 Department of Cardiology, Leiden University Medical Center, Albinusdreef 2, Postal zone 2300 RC, 2333 ZA Leiden, The Netherlands; 2 The Interuniversity Cardiology Institute of the Netherlands, Utrecht, The Netherlands

*This editorial refers to ‘Rationale and design of the Measuring Athlete’s Risk of Cardiovascular events (MARC) Study. The role of coronary CT in the cardiovascular evaluation of middle-aged sportsmen’ by Braber et al. (Neth Heart J. 2014 Nov 20. [Epub ahead of print] doi:10.1007/s12471-014-0630-0)*

The prevalence of coronary artery disease (CAD) in the Dutch population is one of the highest of all diseases and increases with age. Daily physical activity is one way to prevent CAD. Predominantly in men ≥ 45 years, physical exercise is gaining popularity. However, there is an increased risk of acute cardiac events immediately after or during vigorous exercise. This is referred to as the paradox of exercise [1]. In contrast to younger athletes, where exercise-induced cardiac arrest is caused by cardiomyopathies or coronary artery anomalies, in the elderly the main cause of cardiac arrest is CAD. The pathophysiological pathway by which CAD causes cardiac arrest during vigorous exercise is topic of debate. Two pathophysiological mechanisms are possible: (1) Plaque rupture causing myocardial infarction and (2) Demand ischaemia causing ventricular arrhythmias.

Vigorous exercise can induce coronary plaque rupture through several triggering mechanisms: increased wall sheer stress due to high blood pressure or increased heart rate and plaque disruption caused by coronary artery spasms or increased flexing of diseased coronary arteries. Additional risk factors induced by high intensity exercise are the higher sympathetic tone and increased thrombogenicity by increased platelet activation contributing to coronary artery thrombosis after a plaque rupture [2]. A study among 141 men with severe CAD, who died suddenly, compared coronary arteries (with the aid of autopsy) between death during strenuous activity or emotional stress and death at rest. The 25 patients who died during exercise showed significantly more vulnerable plaques and plaque ruptures, compared with the 114 men who died at rest [3].

On the other hand, a recent study found contrasting results. Causes of exercise-induced cardiac arrest and death were examined in 10.9 million middle-aged and elderly men participating in long distance running races. In patients without cardiomyopathy, autopsy reports suggested oxygen mismatch as primary cause in CAD-induced cardiac arrest, instead of acute plaque rupture. At coronary angiography, not one person with serious CAD (31 runners with complete clinical data, 23 died, 8 survivors) had evidence of plaque rupture or thrombus [1].

Currently, in the Netherlands, the medical examination of athletes older than 35 years performing intense exercise consists of medical history, blood testing, and rest and exercise electrocardiogram (ECG). Indeed, exercise ECG is of additional value as it provides information on significant coronary artery stenosis causing ischaemia and gives insight into the patient’s physical condition. However, this method provides no insight into the patient’s risk for plaque rupture.

As proposed in the present study by Braber et al., entitled the Measuring Athlete’s Risk of Cardiovascular events (MARC) study and published in the current issue of the Netherlands Heart Journal [4], coronary computed tomography angiography (CTA) is of high value in determining the total atherosclerotic burden or excluding any atherosclerosis. The prevalence of CAD on coronary CTA has been defined in several populations with chest pain. In the current European Society of Cardiology (ESC) guidelines coronary CTA is indicated for ruling out CAD in symptomatic chest pain patients with low to intermediate likelihood of CAD. However, the prevalence of CAD in the specific setting of asymptomatic elderly athletes is relatively unknown. It is difficult to extrapolate the previously established prevalence to athletes, since vigorous exercise changes the relation between CAD and cardiovascular risk factors. The study by Braber et al. [4] will provide more insight into the prevalence of CAD in these athletes. Additionally, coronary CTA provides important prognostic information for future cardiovascular events [4]. Especially, the prognosis of patients with a normal coronary CTA is excellent, and the event rate increases parallel to increasing stenosis severity. However, it has previously been established that plaque rupture mainly occurs from (vulnerable) non-obstructive lesions. Coronary CTA is currently the only non-invasive diagnostic test that could establish the presence of a vulnerable plaque. As demonstrated, positive coronary vessel remodelling, low attenuation plaques and spotty calcification are associated with subsequent occurrence of acute coronary syndrome [5, 6]. Coronary CTA could be an additional screening tool for vulnerable plaque causing exercise-induced cardiac arrest. A suggestion to Braber et al. is therefore to determine plaque vulnerability beside the coronary artery calcium (CAC) score and luminal stenosis severity. However, obstructive CAD on coronary CTA has a low positive predictive value for myocardial ischaemia. Only approximately half of the ≥ 50 % luminal stenosis on coronary CTA causes myocardial ischaemia [7]. Therefore, for the principle that cardiac events are caused by demand ischaemia, coronary CTA is an inadequate screening method. Especially, the study by Braber et al. [4] includes patients with a normal exercise ECG resulting in a low prevalence of ischaemia in the study population.

A second aim of the study by Braber et al. [4] is to determine the estimated number needed to screen to prevent one cardiovascular event in five years based on a CAC score ≥ 100 and/or ≥ 50 % luminal stenosis on coronary CTA. An event is defined as myocardial infarction, death from CAD, definite angina, probable angina resulting in revascularisation or resuscitated cardiac arrest, stroke, or other atherosclerotic death or other cardiovascular death. The estimated number needed to screen can be calculated by multiplying the number needed to treat (NNT) by the number of persons to screen to find one positive test result. In the proposed study by Braber et al. [4], the NNT is based on a previous sub-study of the JUPITER (Justification for the Use of Statins in Primary Prevention: An Intervention Trial Evaluating Rosuvastatin) trial [8]. Bias will be introduced by using this NNT because the population in this study has different patient characteristics and risk factors. For instance, the patient group in the JUPITER also contained women.

Unfortunately, the patient group will likely be too small to provide information on any relationship between exercise-induced cardiac arrest during follow-up and patients with relevant CAD on CTA (CAC score ≥ 100 and/or luminal stenosis ≥ 50 %). In the study mentioned previously, among 10.9 million marathon runners, the incidence rate was 0.54 per 100,000 participants [1]. Therefore, to fully establish the additional value of coronary CTA for the prevention of exercise-induced cardiac arrest would require large patient cohorts or even randomised trials. It seems unlikely that the proposed study by Braber et al. [4] will be able to provide information on additional prognostic or preventive value.

In conclusion, the proposed study by Braber et al. [4] will provide valuable information about the prevalence of (obstructive) CAD in male athletes ≥ 45 years of age. Nevertheless, due to the low incidence of cardiac arrest, determining additional value in optimising the screening of middle aged and elderly men will be difficult. Furthermore, the low predictive value for myocardial ischemia in patients with a ≥ 50 % stenosis on coronary CTA will not lead to the required information, when we assume that exercise-driven cardiac arrest is the result of oxygen-demand mismatch. A suggestion for an additional image analysis is to score plaque vulnerability as well as plaque characteristics, luminal stenosis and CAC score.

## Disclosures

Michiel A. de Graaf and Alexander R. van Rosendael are supported by a research grant from the Interuniversity Cardiology Institute of the Netherlands (ICIN, Utrecht, the Netherlands). The Department of Cardiology received research grants from Biotronik, Medtronic, Boston Scientific Corporation, St Jude Medical, Lantheus Medical Imaging and GE Healthcare.
